# FTO Prevents Thyroid Cancer Progression by SLC7A11 m6A Methylation in a Ferroptosis-Dependent Manner

**DOI:** 10.3389/fendo.2022.857765

**Published:** 2022-06-03

**Authors:** Fei-Hong Ji, Xing-Hao Fu, Guo-Quan Li, Qi He, Xin-Guang Qiu

**Affiliations:** Thyroid Surgery, The First Affiliated Hospital of Zhengzhou University, Zhengzhou, China

**Keywords:** m6A, ferroptosis, papillary thyroid carcinoma, FTO, SLC7A11

## Abstract

N6 methyladenosine (m6A) modification serves as a novel epigenetic regulatory mechanism that is heavily implicated in the heredity of tumors. Meanwhile, fat mass and obesity-associated protein (FTO) has the potential to affect the regulation of m6A modification in the mRNA of key oncogenes as well as tumor suppressor genes that facilitate tumor progression. In our study, FTO was downregulated in papillary thyroid carcinoma (PTC) tissues. The role of FTO in PTC was assessed by Cell Counting Kit-8 analysis, cell scratch, migration, invasion experiment, flow cytometry apoptosis analysis, and nude mouse experiment. In addition to RNA-Seq and meRIP-Seq, luciferase reporting and mutation analysis have also identified SLC7A11 as the potential FTO regulatory gene. Moreover, X-ray electron microscopy, glutathione (GSH)/oxidized GSH, GPX, malondialdehyde determination, and western blot helped confirmed that FTO inhibited the development of PTC by downregulating the expression of SLC7A11 through ferroptosis. Finally, a rescue experiment was employed to clarify the relationship between FTO and its specific target gene SLC7A11. FTO is able to inhibit the occurrence of PTC by downregulating SLC7A11 in m6A independently, and it functions as a tumor suppressor gene in PTC. These findings could contribute to our understanding of the tumor malignancy regulated by m6A and might lead to new insights for potential biomarkers and therapeutic targets for the treatment of thyroid papillary carcinoma.

## Introduction

In the 1970s, N6-methyladenosine (m6A) modification was detected for the first time (1). For the time being, over 100 epitranscriptome modifications in eukaryotic RNAs (2) have been identified, among which m6A is the most prevalent type (1, 3). m6A modification is generally integrated in shared motif DRACH (D = A, G, U; R = A, G; H = A, C, U), which commonly occurs around stop codons (4).

The m6A modification, catalyzed by a methyltransferase complex, is also termed as the “writer”. A demethylase, also known as the “eraser”, can remove m6A (5, 6). The RNA “reader” protein identifies m6A, which conducts downstream functions when activated (7). The expression of genes encoding writers, erasers, and readers determines the level of m6A (8); therefore, it has been speculated that, in terms of the three types of m6A-related genes, their expression patterns may help assess the risk of diseases associated with m6A dysregulation. During this period, m6A modification can be catalyzed by the methyltransferase complex composed of methyltransferase-like 3 and methyltransferase-like 14; however, fat mass and obesity-associated protein (FTO) and alkB homolog 5 (ALKBH5) may reverse the m6A modifications (4). Through methylated RNA immunoprecipitation and sequencing methods, enriched levels of m6A modifications can be found in 5′-untranslated regions (5′UTRs) and 3′-untranslated regions (3′UTRs) (9), regulating RNA alternative splicing, mRNA translation, and degradation (10).

Increasing evidence has shown the vital role of regulating gene expression played by m6A through affecting mRNA degradation, RNA stability, translation, microRNA maturation, alternative splicing, and cytoplasmic mRNA conversion (2, 11–16), which would be researched under the wide range of normal physiological and pathological conditions, such as metabolism and tumorigenesis (2).

However, abnormal m6A modification is related with tumorigenesis, embryonic developmental disorders, immune cell homeostasis and differentiation failure, and neurological system diseases (17–19). Recently, an increasing number of evidence is found in support of m6A modification to regulate tumor progression, including lung cancer (20), gastric cancer (21), hepatocellular carcinoma (6), glioblastoma (22), and endometrial cancer (23). Therefore, it is worth considering whether m6A also plays a pivotal role in modulating the progression of papillary thyroid carcinoma, yet studies on the role of demethylase FTO in oncogenesis of papillary thyroid carcinoma (PTC) remain rare. The occurrence of tumors may be relevant to both elevated and decreased levels of m6A (24).

FTO is the first identified protein to catalyze the demethylation of m6A (5), mainly localized in the cytoplasm and nucleus (5). FTO is also known for its close correlation with weight gain and obesity in children and adults previously (25, 26). At the molecular level, the knockdown of FTO can enhance m6A enrichment of the 5′UTR and 3′UTR regions in the entire transcriptome. At present, our knowledge of the exact mechanism of FTO in PTC remains insufficient. Finally, the FTO–m6A axis will be explored further in this study to determine whether it may be considered as a new potential therapeutic target for thyroid tumors.

## Materials and Methods

### Human PTC Clinical Tissue Sample

In total, 86 PTC tissues and adjacent non-cancerous tissues were obtained from PTC patients receiving standard resection without radiation therapy at the First Affiliated Hospital of Zhengzhou University from 2020 to 2021. RNA (86 pairs) was separated from frozen tissues for quantitative real-time PCR (qRT-PCR) tests to assess the expression of FTO in PTC, followed by evaluation of clinicopathological parameters through univariate and multivariate Cox regression analyses. The study, carried out in accordance with the Declaration of Principles of Helsinki, won the approval of the Ethics Committee of the First Affiliated Hospital of Zhengzhou University. The license number of the ethical review for the study is 2021-KY-0202-002.

### Cell Cultures

Human papillary thyroid carcinoma cell lines, like TPC-1 and KTC-1 with STR identification, were cultured in 1640 (Gibco, Detroit, MI, USA) in a humid atmosphere of 95% air and 5% CO_2_ at 37°C, which was routinely supplemented with 10% fetal bovine serum (Gibco, Detroit, MI, USA), penicillin (100 units/ml, Grand Island, NY, USA), and streptomycin (100 μg/ml, Grand Island, NY, USA). The STR fingerprint authentications of TPC-1 and KTC-1 cells are available upon request.

### Quantitative Real-Time PCR

A total RNA extraction kit (Vazyme, Nanjing, China) was used to collect total RNA from TPC-1 and KTC-1 cells. The PrimeScript RT reagent kit (Vazyme, Nanjing, China) was employed for the reverse transcription of RNA into cDNA. Then, quantitative real-time PCR was performed through the iTaq Universal SYBR Green Supermix kit (Bio-Rad) and ABI 7500 PCR system (ABI, USA), and GAPDH served as an internal control. Furthermore, all reactions were carried out based on the following cycle parameters: kept at 95°C for 10 min, then followed by 40 cycles of 95°C for 15 s and 60°C for 45 s. Specific amplification through a dissociation curve analysis was conducted. All data represent the average of triplicates under the calculation of 2^−ΔΔCt^ method.

### Western Blot Analysis and Antibodies

Cells were harvested and dissolved in radio immunoprecipitation assay lysis buffer (Beyotime Biotechnology, Jiangsu Province, China), and the protein concentration was assessed by the bicinchoninic acid protein assay (Beyotime Biotechnology, Jiangsu, China). Then, sodium dodecyl sulfate–polyacrylamide gel electrophoresis (NCM Biotech, Jiangsu, China) was put into use for separating proteins, and these were transferred into polyvinylidene difluoride membranes (Millipore, USA) by an electro-blotting apparatus. After blocking with 5% skimmed milk, the membranes were incubated overnight in primary antibodies at 4°C based on the manufacturer’s instructions. The unbound antibody was washed away, followed by further incubation of the membranes with horseradish peroxidase (HRP)-conjugated rabbit secondary antibodies (Beyotime Biotechnology, Jiangsu, China) at room temperature for 1 h. The visualization of bands was performed with an enhanced chemiluminescence detection reagent (Beyotime Biotechnology, Jiangsu, China). All antibodies used in this study were purchased from Abcam (Cambridge, UK). These primary antibodies are known as rabbit monoclonal anti-FTO (ab126605, Abcam), rabbit monoclonal anti-SLC7A11 (ab37185, Abcam), rabbit monoclonal anti-β-actin (ab8226, Abcam), rabbit monoclonal anti-Glutathione Peroxidase 4 (ab125066, Abcam), rabbit monoclonal anti-COX2/Cyclooxygenase 2 (ab179800, Abcam), rabbit monoclonal anti-FACL4 (ab155282, Abcam), rabbit monoclonal anti-Ferritin Heavy Chain (ab183781, Abcam), and rabbit monoclonal anti-NOX1 (ab131088, Abcam).

### Cell Proliferation Assay

Cell proliferation was valued by the Cell Counting Kit-8 (CCK-8) Assay Kit (Vazyme, Nanjing, China). The KTC-1 and TPC-1 cells were cultured in 96-well plates with 1.5 × 10^3^ cells/well; 10 μl of CCK-8 reagent was added to each well and then incubated for 3 h in a dark environment. A microplate reader was used to measure the absorbance at 450 nm. The proportion of relative cell proliferation through the average optical density (OD) of each group was calculated. The formula is as follows: (OD treatment/OD control) × 100%.

### Migration and Invasion Assays

TPC-1 and KTC-1 cells were inoculated into 24-well transwell chambers with polycarbonate membrane (353097) (Corning Life Science, Acton, MA, USA) to evaluate invasion capacities and cell migration under the function of Matrigel (Corning Life Science, 354234) or without it. The upper chamber was injected with cells in a serum-free medium at a density of 4 × 10^5^ cells/well, and the outer chamber got filled with the complete-culture medium. After a 12-h culture process, the cells that adhered to the underside of the chamber for 30 min were fixed with 4% paraformaldehyde, stained with crystal violet for 15 min, and later got calculated under an optical microscope (Zeiss, Germany).

### Lentivirus Vector Infection

Lentiviruses expressing FTO, SLC7A11, FTO-shRNA (target sequences: gcAGCATACAACGTAACTTTG), SLC7A11-shRNA (target sequence: ccCTGGAGTTATGCAGCTAAT), scrambled shRNA, and empty vector were purchased from Genechem Company (Shanghai, China). TPC-1 and KTC-1 cells were selected to establish a stable knockdown and over-expressed cell lines. In brief, 3 × 10^4^ cells were seeded into a 6-well plate and then transfected with the specified lentivirus using HitransG P/A (Genechem, China), with reference to the manufacturer’s instructions. In addition, infected cells were selected by using 2.5 μg/ml puromycin (MCE, USA) for ≥14 days, and the transfection efficiency was verified by RT-qPCR and Western blot analysis.

### Methylated RNA Immunoprecipitation Sequencing and Data Analysis

Seqhealth Technology Co., Ltd. (Wuhan, China) was the main supplier of MeRIP sequencing and following data analysis. The stably transfected cell, TPC-1 cells with FTO over-expression, and controlled cells with empty vector were collected. Total RNAs were extracted by TRIzol Reagent (Invitrogen, catalog number 15596026). After RNA extraction with the help of DNaseI, DNA digestion was performed. Then, the RNA quality was evaluated by checking A260/A280 with Nanodrop TM OneC spectrophotometer (Thermo Fisher Scientific Inc.). RNA integrity was verified by 1.5% agarose gel electrophoresis. In the end, qualified RNAs were weighed by Qubit3.0 with QubitTM RNA Broad Range Assay kit (Life Technologies, Q10210). Furthermore, 50 μg total RNAs was used for polyadenylated RNA enrichment by VAHTS mRNA Capture Beads (VAHTS, catalog number N401-01/02). Moreover, 20 mM ZnCl2 was added to mRNA and incubated at 95°C for 5–10 min until the RNA fragments were evenly distributed in 100–200 nt. Then, 10% RNA fragments was saved as “input”, while the remaining parts were utilized for m6A immunoprecipitation (IP). The specific anti-m6A antibody (Synaptic Systems, 202203) was applied into m6A immunoprecipitation. TRIzol reagent (Invitrogen, catalog number 15596026) was utilized to prepare both input and IP RNA samples. In addition, referring to the manufacturer’s instructions, the stranded RNA sequencing library was established by KC-Digital TM Stranded mRNA Library Prep Kit for Illumina^®^ (catalog number DR08502, Wuhan Seqhealth Co., Ltd., China). The kit used a unique molecular identifier (UMI) of 8 random bases to label pre-amplified cDNA molecules, thereby removing repetitive bias in PCR and sequencing steps. The library products with 200–500 bp were enriched, quantified, and finally sequenced on Novaseq 6000 sequencer (Illumina) with the PE150 model. In a word, the raw data was aligned with human genome GRCh37/hg19 *via* the STAR software (version 2.5.3a). Then, m6A peaks were acquired by the ExomePeak software (version 3.8) based on the Ensembl database. Sequence motifs abundant in m6A peak regions were identified using Homer (version 4.10). Gene Ontology (GO) analysis and Kyoto Encyclopedia of Genes and Genomes (KEGG) enrichment analysis were both implemented by KOBAS software (version: 2.1.1) for annotated genes, and the cutoff value of the corrected *p*-value at 0.05 aimed at assessing the statistical significance of enrichment.

### RNA-seq and Data Analysis

UID RNA-seq experiment, high throughput sequencing, and data analysis were performed by Seqhealth Technology Co., Ltd. (Wuhan, China). TRIzol Reagent (Invitrogen, catalog number 15596026) was used for the extraction of total RNA from TPC-1-Lv-FTO/TPC-1-vector. DNA digestion was carried out after RNA was extracted with the help of DNaseI. RNA quality was valued by studying A260/A280 with Nanodrop TM OneCspectrophotometer (Thermo Fisher Scientific, Inc.). Later, RNA integrity was tested by 1.5% agarose gel electrophoresis. The qualified RNAs were quantified by Qubit3.0 equipped with QubitTM RNA Broad Range Assay kit (Life Technologies, Q10210). Then, total RNAs weighing 2 μg were used for the library preparation of stranded RNA sequencing using the KC-DigitalTM Stranded mRNA Library Prep Kit for Illumina^®^ (catalog number DR08502, Wuhan Seqhealth Co., Ltd., China) based on the manufacturer’s instruction. The kit used a UMI of 8 random bases to label pre-amplified cDNA molecules, hereby eliminating repetitive bias in PCR and consequent steps. The library products with 200–500 bp were enriched, quantified, and finally sequenced on the Novaseq 6000 sequencer (Illumina) under the PE150 model. Distinctively expressed genes were used to draw heat maps and conduct the KEGG ontology enrichment analysis. As for the KEGG enrichment analysis, *P*-value <0.05 was used as the criteria of a significantly enriched gene set. NCBI (http://www.ncbi.nlm.nih.gov/Genbank/GenbankSearch.html) and KEGG databases (http://www.genome.jp/kegg/) got respectively used for analyzing gene ontology (GO) and signal pathways.

### MeRIP-qPCR

Overall, 2 μg of total RNA and m6A spike-in control mixture was added to 300 μl of 1IP buffer (50 mM Tris-HCl, pH 7.4, 150 mM NaCl, 0.1% NP40, and 40 U/μl RNase inhibitor) containing 2 μg of anti-m6A rabbit polyclonal antibody (Synaptic Systems). Twenty microliters of Dynabeads™ M-280 sheep anti-rabbit IgG suspension of each sample was blocked with freshly prepared 0.5% bovine serum albumin for 2 h at 4°C, washed three times with 300 μl of 1IP buffer, and resuspended in the total RNA–antibody mixture prepared as detailed above. Binding of RNA to m6A-antibody beads was head-to-tail rotated for 2 h at 4°C, and the enriched RNA was washed with 200 μl elution buffer for 1 h at 50°C, after which the co-precipitated RNA was isolated, and the RNA was subjected to qRT-PCR.

### m6A RNA Methylation Quantification

The m6A quantification was carried out utilizing the EpiQuik™ m6A RNA Methylation Quantification Kit (Colorimetric) (P-9005, Epigentek, China). The relevant solutions were added to each well according to the manufacturer’s instructions. The solution was mixed by gentle tilting from side to side, after which m6A RNA capture was performed. It was ensured that any residual wash buffer in the wells was thoroughly removed at each wash step. Signals were detected at the end when the color of the positive control wells changed to medium blue. A 100-μl Stop Solution was then added to each well to stop the enzyme reaction. After adding the Stop Solution, the color of the compound solution changed to yellow. The absorbance was read at 450 nm on a microplate reader.

### Immunohistochemistry

The human tissue sections need to be deparaffinized and rehydrated, and 10 mM citrate buffer should be used for antigen extraction. After treatment with 3% H_2_O_2_, the sections were soaked with the primary antibodies overnight at 4°C and then incubated with the HRP-conjugated secondary antibody for 1 h at room temperature. Referring to the manufacturer’s instructions, diaminobenzidine (G1212-200T) (Wuhan Servicebio, China) was used to check the immune complexes. The Image-Pro Plus software (Media Cybernetics, MD, USA) was chosen to quantify protein levels by calculating the integrated optical density of each stained area. In short, the sections were deparaffinized and boiled in Tris antigen retrieval buffer. Afterwards, the sections were incubated overnight with primary antibodies against FTO (ab126605, Abcam, 1:500 dilution), and SLC7A11 (ab37185, Abcam, 1:400 dilution) at 4°C. Immunohistochemistry images were captured by using an Axio Imager 2 (Zeiss). FTO and SLC7A11 signals in immunohistochemical staining were assigned with values by a semi-quantitative scoring system based on the product of positive percentage and signal intensity. The positive percentage formula is as follows: 0 = 0%, 1 = 1–25%, 2 = 26–50%, 3 = 51–75%, 4 = above 75%. On the other hand, the signal intensity is as follows: 0 = no staining, 1 = weak staining, 2 = medium staining, 3 = strong staining. Then, the median value of FTO and SLC7A11 scoring in all specimens got selected as the cutoff value. Those with FTO or SLC7A11 scoring below the cutoff value were labeled as the low-expression specimen, while for those scoring higher than the value, they were labeled as the high-expression specimen. Two trained pathologists performed the quantification of IHC staining.

### Apoptosis Assay

Cells were stained by using Annexin V Alexa Fluor 647/PI Apoptosis detection Kit (4A Biotech Co., Beijing, China). Referring to the manufacturer’s recommendations, apoptosis was analyzed by the BD Accuri C6 Flow cytometer (BD Biosciences, USA). The cells, first digested with 0.25% trypsin (without EDTA), were then washed twice with phosphate-buffered saline (PBS; centrifugation at 1 × 10^3^ rpm for 5 min), and 1 × 10^6^ cells were collected. The re-suspension of cells was performed in 1 × 10^3^ µl binding buffer. As 100 μl of cell suspension in a 5-ml flow tube was taken out, 5 μl Annexin V/Alexa Fluor 647 was added then. The suspension was incubated at room temperature for 5 min in dark experimental conditions, added with 10 μl of 20 ug/ml propidium iodide solution and 400 μl PBS through the use of a flow cytometer to collect 2 ×10^4^ events. Apoptotic cells in their early and late stages were separated and then presented in a quadratic graph.

### Animal Experiments

Male BALB/c nude mice aged between 5 and 6 weeks were purchased from Beijing Vital River Laboratory Animal Technology (Vital River, Beijing, China) and kept in an animal facility free from pathogen for at least 7 days before use. Then, a cohort of 15 male nude mice was assigned to the groups of TPC-1-Lv-FTO, TPC-1-vector, and TPC-1 (*n* = 5 per group) under random selection. A total of 1 × 107 cells kept in a mixture of 100 μl PBS and Matrigel were injected into the BALB/c nude mice subcutaneously. Tumor volumes were then monitored twice per week after the end of the first week. After 4 weeks, the tumor tissues of sacrificed mice were collected for subsequent analysis. The entire experiment was approved by the Animal Care and Use Committee of the First Affiliated Hospital of Zhengzhou University.

### Luciferase Reporter Assays and Mutagenesis Assay

The dual-luciferase reporter gene vector of target gene SLC7A11 and mutants with mutations in the FTO-binding site were formulated respectively as Luc-SLC7A11-3’UTR and Luc-SLC7A11-3’UTR-MUT. Two reporter plasmids, FTO and FTO-NC plasmids, were co-transfected into 293T cells, and the cells were lysed for 36 h after transfection and centrifuged at 12,000 rpm for 1 min. Next, the supernatant was collected, and Dual-Luciferase^®^ Reporter Assay System (Promega) was used to detect luciferase activity. Furthermore, 100 μl of firefly luciferase working solution was added into each cell sample to detect firefly luminescence (firefly luminescence), and Renilla luciferase working solution was added into the sample to identify Renilla luciferase (renilla luciferase). It is worth noting that luciferase was employed as well. The experiment was repeated three times.

### Transmission Electron Microscopy

Firstly, TPC-1-Lv-FTO cells were collected and placed in 2.5% glutaraldehyde phosphate (0.1 M, pH 7.4; Servicebio, Wuhan, China) overnight at 4°C, then fixed in 2% buffered osmium tetraoxide, and finally embedded in Epon812 (Merck), followed by dehydration. Then, ultrathin sections were cut out (60 nm in thickness), and uranyl acetate and lead citrate were used for staining. Eventually, a TEM microscope was utilized for picture checking (FEI, Hillsboro, OR, USA).

### Assessment of the Glutathione/Oxidized GSH Ratio

Cellular glutathione **(**GSH)/oxidized GSH (GSSG) ratio was analyzed by using the GSH and GSSG assay kit (Beyotime Biotechnology, Jiangsu, China, S0053) under the manufacturer’s instructions. Full sample preparation, standard solution preparation, and concentration detection were carried out by referring to the manufacturer’s protocols. Fresh cells were used for the assay, and PBS was employed to wash the cells once. The cells were collected by centrifugation, and the supernatant was aspirated, with a protein removal reagent added to the cell pellet. Liquid nitrogen was used under 37°C water bath to quickly freeze, and the sample was thawed twice. The cells were placed at 4°C or ice bath for 5 min and harvested by centrifugation at 1 × 10^4^ g for 5 min at 4°C. Then, the supernatant was taken out, and it was time to determine the amount of total glutathione (GSSG + GSH) by using a plate reader to measure the absorbance at 412 nm. The use of appropriate reagents to remove GSH in the sample was first done, and then the above-mentioned reaction principle was employed to determine the content of GSSG. The content of GSSG was subtracted from the amount of total glutathione (GSSG + GSH) to calculate the content of GSH.

### Measurement of MDA Content

Based on the manufacturer’s instructions, Lipid Peroxidation MDA Assay Kit (Beyotime Biotechnology, Jiangsu, China, S0131S) was used to make a quantitative detection of malondialdehyde (MDA) in the cells by the colorimetric method to analyze the level of lipid oxidation in the sample. The cell samples were lysed using cell lysis buffer. After lysis, the samples were centrifuged at 12,000*g* for 10 min at 4°C for the subsequent determination of supernatant. The MDA detection working solution was added to the sample supernatant, mixed well, and heated at 100°C or under the condition of boiling water bath for 15 min. The mixtures were then cooled down in a water bath till room temperature and centrifuged at 1,000 *g* for 10 min at room temperature. Two hundred microliters of supernatant was later taken out and added to a 96-well plate, and then its absorbance at 532 nm was measured by a microplate reader.

### Iron Load Assay

Referring to the manufacturer’s instructions, the iron colorimetric assay kit (Applygen, Beijing, China, E1042) was used to quantitatively detect and analyze Fe^2+^ in the cells under the ferrozine colorimetric method. The cells cultured in the iron-containing medium were washed twice with cold PBS, then the PBS got aspirated, and the cell sample was lysed with cell lysate and placed on a shaker for 2 h. in total, 4.5% potassium permanganate was added, mixed uniformly, and incubated at 60°C for 1 h. After the sample was cooled down to room temperature, iron ion detection reagent was well added and mixed, followed by incubation at room temperature for 30 min. Next, 200 μl of supernatant was taken and added to a 96-well plate; then, its absorbance at 550 nm was measured with a microplate reader.

### Measurement of GPx Assay

With reference to the manufacturer’s instructions, Cellular Glutathione Peroxidase Assay Kit with a kit for glutathione peroxidase (GPx) activity in tissues or other samples, NADPH (Beyotime Biotechnology, Jiangsu, China, S0056) got employed for cell detection. The cells were collected through a cell scraper and washed once with PBS. The cell sample, lysed with cell lysate, was then centrifuged at 12,000 *g* for 10 min at 4°C. The supernatant for identifying the enzyme activity was taken out. In the next stage, on a 96-well plate, the detection buffer was added to test the sample, and the GPx detection working solution in sequence was evenly mixed and incubated at room temperature for 15 min. Next, after the adding and mixing of peroxide reagent solution, attached with the appropriate enzyme label, the solution was put immediately at 25°C and room temperature. The measurement of A340 with an instrument or a trace ultraviolet spectrophotometer was conducted for 5 consecutive minutes.

### Statistical Analysis

Statistical analysis was performed using GraphPad Prism 8.0 (GraphPad, Inc., USA) and SPSS 22.0 (SPSS, Inc., USA) software. All statistical tests followed the two-way path, and *p <*0.05 was treated with statistical significance. The difference between groups was tested by one-way analysis of variance. Representative data was demonstrated as mean ± SD. In addition, a correlation analysis of gene expression was carried out with linear regression. The *P*-values for every result were labeled on the figures, and *P <*0.05 was reckoned as statistically significant (*P <0.05, **P <0.01, ***P <0.001, ****P <0.0001).

## Results

### FTO Expression Is Downregulated in Patients With Papillary Thyroid Carcinoma

**Figure 1:**
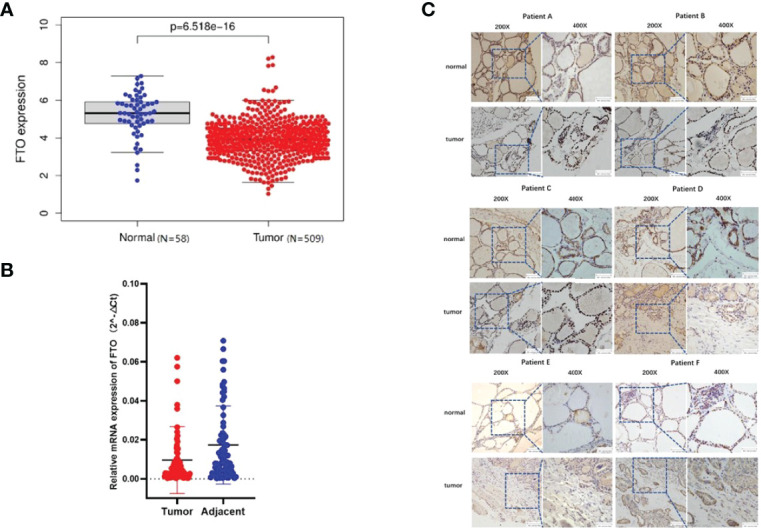
Fat mass and obesity-associated protein (FTO) expression is downregulated in patients with papillary thyroid carcinoma (PTC). **(A)** In The Cancer Genome Atlas database, the expression of FTO in patients with papillary thyroid carcinoma and in normal people. **(B)** Expression of FTO in 86 pairs of PTC specimens as determined by RT-qPCR. **(C)** Representative images of immunohistochemistry staining for FTO in specimens from patients with PTC.

After The Cancer Genome Atlas (TCGA) data set (http://ualcan.path.uab.edu/index.html) was studied, the analysis of PTC cohort from TCGA has shown that FTO was downregulated in tumor tissues compared with the corresponding noncancerous tissues (**Figures 1A, B**).

To estimate FTO expression in PTC and the probable clinical significance, 86 cases of tumor and adjacent tissue from PTC patients were analyzed (**Table 1**). As a result, real-time qRT-PCR showed a remarkable reduction in FTO mRNA levels in PTC tissues in comparison with non-cancerous controls. Immunohistochemistry staining displayed the existence of a number of positively FTO-stained cells in normal thyroid control tissues, while negative staining was detected in PTC tissues (**Figure 1C**). GAPDH got employed as internal control, individually, in the qRT-PCR assays (*P < 0.05, **P < 0.01, and ***P < 0.001).

**Table 1:** Correlation between fat mass and obesity-associated protein (FTO) expression and the clinicopathological features of papillary thyroid carcinoma.

Characteristics		Number of patients	FTO expression		*p*
			Low	High	
Age					
<45		50	40	10	0.09587
≥45		36	23	13	
Gender					
Female		65	46	19	0.3594
Male		21	17	4	
Stage					
Stage 1		76	57	19	0.31371
Stage 2		10	6	4	
Stage 3		0	0	0	
Stage 4		0	0	0	
T					
T1a		49	37	12	0.68968
T1b		30	20	10	
T2		6	5	1	
T3		1	1	0	
N					
N0		62	48	14	0.02707
N1a		23	14	9	
N1b		1	0	0	
M					
M0		86	63	23	
M1		0	0	0	

P <0.05 indicates a significant relationship among the variables.

### FTO Inhibits PTC Proliferation, Migration, and Invasion *In Vitro* and *In Vivo*

**Figure 2:**
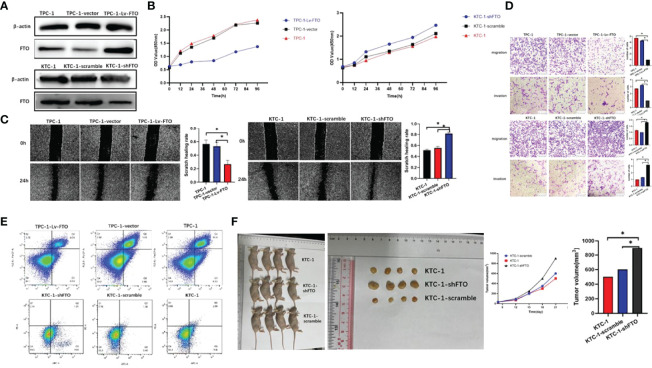
Fat mass and obesity-associated protein (FTO) inhibits papillary thyroid carcinoma (PTC) proliferation, migration, and invasion *in vitro* and *in vivo*. **(A)** Construction of the FTO overexpression model of TPC-1 and the FTO silencing model of KTC-1 as verified by western blot. **(B)** Cell proliferation was applied to evaluate the proliferation abilities of three PTC cell lines with knockdown or overexpression of FTO. **(C)** Wound healing assays showed the suppressed migration of PTC cells, with knockdown or overexpression of FTO, respectively, following FTO overexpression. In contrast, FTO knockdown promoted the migration (wound closure) of PC cells. **(D)** Transwell migration and invasion experiment. **(E)** Overexpression of FTO significantly increased cell apoptosis in PTC cells by fluorescence-activated cell sorting. **(F)** Macroscopic image of the tumor size in a xenograft mouse model in which the mice were injected with control and KTC-1-shFTO cells (stably knockdown FTO). The knockdown of FTO dramatically promoted PTC tumor growth in an orthotopic xenograft mouse model. *, P<0.05.

To evaluate the functional roles of FTO in PTC, the expression of FTO was first identified in PTC cell lines—TPC-1 cells which were chosen to establish FTO-silencing and FTO-overexpression models. Consequently, the transfection efficiency was verified by western blot (**Figure 2A**).

Indications from the CCK-8 assays showed that the upregulation of FTO weakened the proliferation capability of TPC-1 cells, while FTO silencing proved the opposite effect in KTC-1 cell (**Figure 2B**). Besides this, the FTO knockdown effects on the proliferation and migration of PTC cells were verified using a scratch-wound model. Then, these results demonstrated that FTO knockdown significantly promoted the gap closure rate of PTC cells (**Figure 2C**). Transwell assays were conducted, and the inhibition of FTO was found to facilitate both the migration and invasion of PTC cells. In addition, the over-expression of FTO can impair these phenotypes (**Figure 2D**). On the whole, these findings indicate that increased FTO expression may curb the malignancy of PTC cells. The flow cytometry experiments suggest a stronger apoptotic ability in FTO knockout cell lines, while FTO overexpression cell lines show the opposite trend (**Figure 2E**).

In order to further verify the role of FTO in PTC, *in vivo* experiments with subcutaneous tumor models were implemented. On top of that, when FTO was over-expressed, the volumes and weights of the xenografted tumors decreased compared with those of the control group. These results indicate that FTO would exert an inhibitory effect on PTC tumor growth in *in vivo* and *in vitro* experiments. In addition, the xenografts derived from TPC-1-Lv-FTO cells (FTO overexpression) demonstrated tumor weight and volume which were much lower than those extracted from TPC-1 vector (empty) (**Figure 2F**). The monitoring of the size and the weight of the tumor formed in the subcutaneously implanted mice model was performed every 3 days (*P < 0.05, **P < 0.01).

### FTO Effects on the Transcription Profile of PTC Cells by RNA-seq

**Figure 3:**
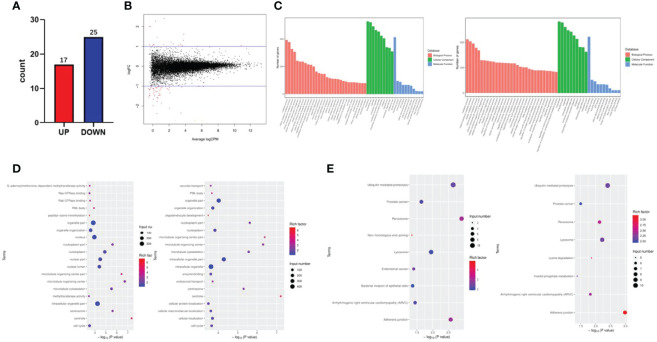
Fat mass and obesity-associated protein (FTO) effects on the transcription profile of papillary thyroid carcinoma (PTC) cells by RNA-seq. **(A, B)** A volcano plot shows the statistically upregulated (red) and downregulated (blue) genes between PTC-1-vector cells (control) and PTC-1-Lv-FTO cells (FTO overexpression), logFC <-1 or logFC >1, *p* 0.05, false discovery rate <0.05. **(C)** Signal pathways related to upregulated and downregulated genes reveal the role of differentially expressed genes in signal transduction. **(D)** The Kyoto Encyclopedia of Genes and Genomes analysis shows that FTO-over-expressed regulatory pathways are involved in cell proliferation, cell cycle, and apoptosis. **(E)** Gene ontologies related to the upregulated and downregulated genes between PTC-1-vector cells (control) and PTC-1-Lv-FTO cells (FTO overexpression).

To further investigate the potential pathways of the FTO tumor suppressor effect in papillary thyroid carcinoma, RNA-seq sequencing on TPC-1-Lv-FTO and vector cell lines was performed and repeated 3 times. Through RNA-seq sequencing, 17 genes were found to be upregulated, while 25 genes were downregulated in the presence of TPC-1-Lv-FTO and vector cell lines [logFC <-1 or logFC >1, *p <*0.05, false discovery rate (FDR) <0.05] (**Figures 3A, B**, **Tables 2**, **3**). This result is shown through a volcano plot (**Figure 3B**). The gene enrichment pathway of RNA-seq correlated with the FTO expression (**Figure 3C**), and the GO (**Figure 3D**) and KEGG pathways (**Figure 3E**) of the upregulated genes and downregulated genes were analyzed respectively.

**Table 2:** A total of 25 genes were found to be downregulated in TPC-1-Lv-FTO and vector cell lines by RNA-seq sequencing [logFC <-1, *p* < 0.05, false discovery rate (FDR) <0.05].

Gene	*P*-value	FDR	logFC
SLC7A11	0.009096	0.047954	-1.04876
CHST9	0.000896	0.041327	-1.09716
HOGA1	0.000844	0.04005	-1.48277
RP11-422P24.9	0.000791	0.038368	-1.77272
DNAH1	0.000708	0.035441	-1.55712
FXYD2	0.000638	0.033355	-1.44384
RP11-539I5.1	0.000627	0.033355	-1.07863
FER1L4	0.000553	0.03103	-1.35845
H1F0	0.000527	0.030097	-1.04298
FXYD6-FXYD2	0.000371	0.024499	-1.60873
HLF	0.000335	0.022739	-1.30797
FAT2	0.000212	0.017259	-1.48293
THEMIS2	0.000206	0.01705	-1.13356
CHDH	0.000166	0.015202	-1.26341
NDRG2	0.000104	0.010884	-1.2218
C15orf59	0.0001	0.01077	-1.46561
RGAG4	9.78E-05	0.010698	-1.46147
RASAL1	3.60E-05	0.005089	-1.9797
PKP2	3.39E-05	0.004976	-1.02555
PAX5	1.87E-05	0.003395	-1.34244
BGN	1.20E-05	0.00238	-1.18602
PPFIBP2	3.02E-06	0.000897	-1.08143
GRAMD1B	1.88E-06	0.000604	-1.05182
TP73-AS1	5.14E-07	0.000198	-1.85523
TENM2	2.92E-09	3.46E-06	-2.11466

**Table 3:** A total of 17 genes were found to be upregulated in TPC-1-Lv-FTO and vector cell lines by RNA-seq sequencing [logFC >1, *p* < 0.05, false discovery rate (FDR) <0.05].

Gene	*P*-value	FDR	logFC
ZCCHC5	0.000911	0.041571	1.00622
CLEC4E	0.000769	0.037823	1.993987
ZNF300	0.000101	0.01077	1.104633
SERPINE1	5.60E-05	0.007271	1.613194
ABCB4	5.48E-05	0.007259	1.45777
LONRF3	4.37E-05	0.006016	2.021062
KCNK3	3.83E-05	0.005325	1.059183
RP11-334L9.1	2.06E-05	0.003421	1.129863
RELN	1.27E-05	0.002446	1.213992
CH507-513H4.1	6.02E-06	0.001401	2.274332
NCAM1	4.59E-06	0.001181	1.305366
BIRC3	1.43E-06	0.000481	1.06276
RP11-39C10.1	1.72E-07	7.19E-05	1.005149
INHBA	6.19E-08	3.18E-05	1.11786
ZNF804A	3.51E-09	3.54E-06	2.406264
FTO	3.10E-10	4.79E-07	1.001468
HSPA5	4.12E-14	6.35E-10	1.044672

### FTO Effects on the Transcription Profile of PTC Cells by meRIP-Seq

**Figure 4:**
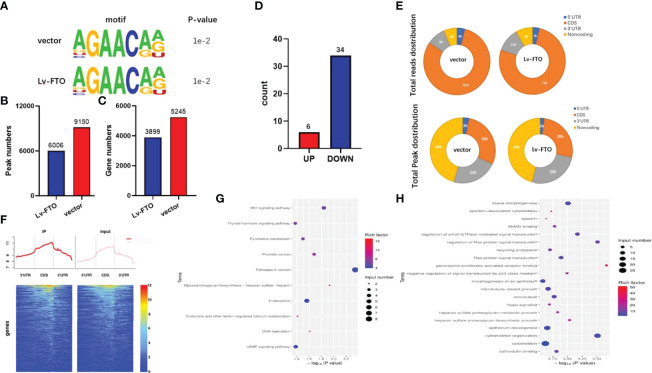
FTO effects on the transcription profile of papillary thyroid carcinoma (PTC) cells by meRIP-Seq. **(A)** The motif analysis performed using the HOMER program showed that “GAACA” is the m6A consensus motif of TPC-1-vector and TPC-1-Lv-FTO cells. **(B, C)** m6A-seq measures the number of m6A peaks and genes in TPC-1 cells. **(D, E)** Graphs of m6A peaks and reads distribution illustrating the proportion of common m6A peaks in the indicated regions in TPC-1 cells. **(F)** Distribution map (and heat map) of reads in the peak gene functional area. **(G)** Compared to PTC-1-vector cells, Kyoto Encyclopedia of Genes and Genomes analysis of m6A methylation downregulated genes in the m6A sequencing of PTC-1-Lv-FTO cells. **(H)** Compared to PTC-1-vector cells, gene ontology analysis of m6A methylation downregulated genes in the m6A sequencing of PTC-1-Lv-FTO cells.

After the comparative RNA-Seq analysis of FTO over-expressed TPC-1 cells (TPC-1-Lv-FTO) and control group (TPC-1-vector), a decreased pattern was detected in the Lv-FTO cells of m6A levels. Next, to map the m6A modifications, m6A Seq was carried out. The motif analysis performed by using the HOMER program has shown that “GAACA” serves as the m6A consensus motif of TPC-1 cells (**Figure 4A**). Further investigations on the distribution of the peak m6A indicated that m6A in the vehicle group and the Lv-FTO group showed a similar total distribution pattern. In order to discover the precise mechanisms in support of the observed FTO-dependent phenotypes, an integral approach combining meRIP-Seq and RNA-Seq was employed through the utilization of vector cells and stable FTO’s over-expression. In general, m6A-seq identified 5,245 and 3,899 gene numbers from 9,150 and 6,006 m6A peaks in vector (normal control) and Lv-FTO cells (**Figures 4B, C**). Through meRIP-Seq sequencing, 6 genes were found to be upregulated, while 34 genes were downregulated in the presence of TPC-1-Lv-FTO and vector cell lines (logFC <-1 or logFC >1, *p <*0.05, FDR <0.05) (**Tables 4**, **5**, and **Figures 4D, E**). meRIP-Seq has shown m6A peaks, and the read distribution demonstrates the proportion of common m6A peaks in the indicated areas in TPC-1 cells and the distribution of reads in the peak of the gene functional area (**Figure 4F**). The demethylated genes in TPC-1-Lv-FTO (logFC >1, *p <*0.05) were screened through meRIP-Seq and analyzed with GO (**Figure 4G**) and KEGG (**Figure 4H**).

**Table 4:** A total of 34 genes were found to be downregulated in TPC-1-Lv-FTO and vector cell lines by meRIP-Seq sequencing [logFC <-1, *p* < 0.05, false discovery rate (FDR) <0.05].

Gene	logFC	*P*-value	FDR
EEA1	-5.53104	1.78E-36	9.87E-34
MORC2	-4.93912	8.58E-24	4.48E-21
PLCE1	-4.40551	4.35E-16	2.05E-13
SRSF8	-4.33839	7.47E-21	3.70E-18
RNF111	-4.08358	4.87E-15	2.18E-12
FBXO38	-3.94607	1.68E-13	6.87E-11
ATP13A3	-3.4315	2.31E-11	8.04E-09
SLC7A11	-3.268	2.43E-09	7.37E-07
VANGL1	-3.09663	1.33E-12	5.21E-10
THTPA	-2.86034	6.12E-09	1.75E-06
ADAMTS12	-2.82649	4.15E-08	1.12E-05
C15orf52	-2.79407	0.000122	0.022122
NKX3-1	-2.79407	1.28E-09	4.01E-07
GPR176	-2.6853	7.00E-07	0.000165
POLE4	-2.62415	1.16E-11	4.22E-09
IER5L	-2.60143	1.96E-10	6.36E-08
ARHGAP35	-2.57168	5.93E-09	1.74E-06
KIF14	-2.52408	2.80E-08	7.75E-06
ARHGAP35	-2.45303	5.85E-07	0.000141
EPB41	-2.40551	1.81E-05	0.003704
RC3H2	-2.32459	1.13E-07	2.88E-05
ZNFX1	-2.20911	4.11E-07	0.000102
NCOA6	-2.09663	2.18E-06	0.00049
CAMSAP1	-2.05281	3.79E-06	0.000811
ARID1B	-2.04165	2.18E-06	0.000501
MBTPS1	-2.01646	2.30E-06	0.000504
SPECC1L	-1.99047	6.65E-06	0.001391
CHID1	-1.89841	0.000178	0.03153
NDST1	-1.84911	1.90E-05	0.003797
RABEP1	-1.83876	3.32E-05	0.006511
DNAJB4	-1.79407	9.57E-05	0.018022
PHLDB2	-1.75225	9.57E-05	0.018022
SNPH	-1.71607	0.00027	0.046228
USP54	-1.56062	0.000242	0.042181

**Table 5:** Six genes were found to be upregulated in TPC-1-Lv-FTO and vector cell lines by meRIP-Seq sequencing [logFC >1, *p* < 0.05, false discovery rate (FDR) <0.05].

Gene	logFC	*P*-value	FDR
ZNF235	6.335212	6.24E-39	3.67E-36
JARID2	4.595213	6.59E-12	2.48E-09
RNPS1	4.10336	7.70E-08	2.01E-05
ZNF155	3.401389	7.46E-14	3.19E-11
ZNF114	3.138355	1.10E-10	3.71E-08
YWHAE	2.416496	0.000113	0.0208

### RNA-seq and meRIP-Seq Combined With RNA-Seq Reveals SLC7A11 as a Target of FTO

**Figure 5:**
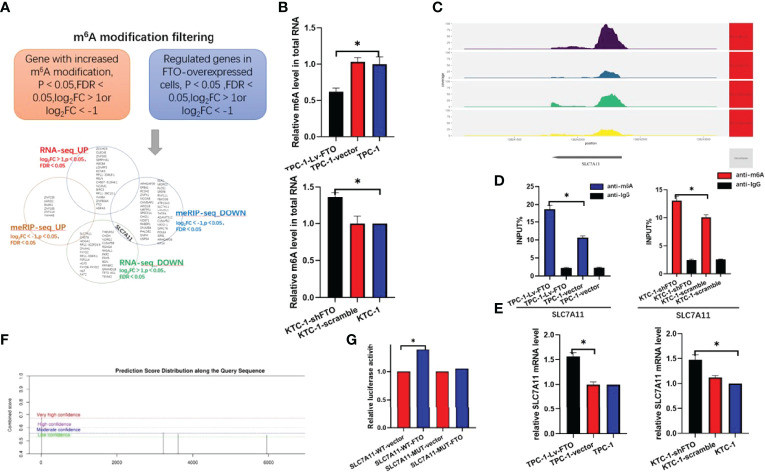
RNA-seq and meRIP-Seq combined with RNA-Seq reveals SLC7A11 as a target of Fat mass and obesity-associated protein (FTO). **(A)** Screening SLC7A11 as the downstream target of FTO by bioinformatics analysis. m6A site of SLC7A11 in genetic coverage. **(B)** EpiQuik™ m6A RNA Methylation Quantification Kit was used to identify whether m6A modification was enriched in the TMBIM6 sequence. **(C)** m6A peak in SLC7A11 by meRIP-Seq sequencing. **(D)** MeRIP assays were performed to identify variations in m6A modification enrichment in SLC7A11 after silencing or overexpressing FTO in lung squamous cell carcinoma cells. **(E)** qRT-PCR analysis of SLC7A11 mRNA after FTO inhibition or overexpression. **(F, G)** The luciferase report experiment confirmed that the overexpression of FTO eliminated the m6A modification of SLC7A11 mRNA in TPC-1 cells. *, P<0.05.

Upon combination analysis of meRIP-Seq and RNA-Seq and selection of SLC7A11, it is speculated that SLC7A11 may be used as a downstream target gene of FTO to regulate papillary thyroid carcinoma (**Figure 5A**).

First, we first verified whether FTO altered the m6A levels in papillary thyroid cancer cell lines, and we found that TPC-1-Lv-FTO occurred with the downregulation of m6A levels, while KTC-1-shFTO occurred with the upregulation of m6A levels (**Figure 5B**). By checking the meRIP-seq data to determine whether the m6A modification was enriched in the SLC7A11 sequence, we found that SLC7A11 clearly showed m6A level downregulation by comparing the difference in m6A peak on the SLC7A11 INPUT and IP samples (**Figure 5C**).

Next, we performed anti-m6A immunoprecipitation and use qRT-PCR to analyze the m6A-immunoprecipitated RNA. The results showed that FTO overexpression resulted in the downregulation of m6A modification of SLC7A11 mRNA. In contrast, FTO knockdown increased the m6A modification of SLC7A11 mRNA (**Figure 5D**). From the perspective of SLC7A11 mRNA, the mRNA level of SLC7A11 was down- or upregulated when FTO was over-expressed or knocked down (**Figure 5E**). These results fully illustrate the regulation of SLC7A11 expression by FTO.

In order to prove the essential role of m6A in regulating SLC7A11, a luciferase reporter, an inserted mutant (Mut), and a wild-type (WT) SLC7A11–3′UTR sequence equivalent to its mutating putative m6A sites were designed (**Figure 5F**). The mutation of m6A consensus sequences were generated by substituting adenine for thymine. Then, the relative luciferase activities of the wild type and 5 mutant SLC7A11 3′UTR reporter vectors in TPC-1-Lv-FTO (overexpression) cells were implemented and evaluated. The dual-luciferase reporter experiment of the over-expression of FTO increased the luciferase activity of the reporter carrying the wild-type SLC7A11 3′UTR rather than its 3′UTR mutant in the FTO recognition site, demonstrating that FTO could be bound to the putative m6A sites of SLC7A11 3′UTR (**Figure 5G**).

### SLC7A11 Is Identified as an Oncogenic Driver in PTC

**Figure 6:**
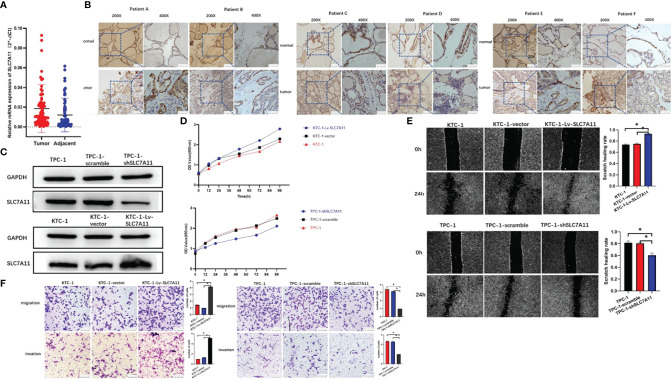
SLC7A11 is identified as an oncogenic driver in papillary thyroid carcinoma (PTC). **(A)** Expression of SLC7A11 in 86 pairs of PTC specimens as determined by RT-qPCR. **(B)** Representative images of immunohistochemistry staining for SLC7A11 in specimens from patients with PTC. **(C)** SLC7A11 protein expression in control and KTC-1-Lv-SLC7A11/TPC-1-shSLC7A11 cells was assessed by western blot. **(D)** CCK-8 assays to analyze cell viability in control and KTC-1-Lv-SLC7A11 and TPC-1-shSLC7A11 cells. **(E)** Scratch assay. SLC7A11 knockdown significantly increased PTC cell death and attenuated the migration ability *in vitro*. **(F)** *In vitro* migration and invasion of control and KTC-1-Lv-SLC7A11/TPC-1-shSLC7A11 cells as assessed by transwell assay. *, P<0.05.

In order to examine the expression of SLC7A11 in PTC and its potential clinical significance, 86 cases of tumor and corresponding noncancerous tissue from PTC patients were evaluated. As a result, real-time qRT-PCR showed a crucial elevation in SLC7A11 mRNA levels in PTC tissues compared with those under noncancerous controls (**Figure 6A**). Immunohistochemistry staining has demonstrated masses of positively SLC7A11-stained cells in PTC tissues, while negative staining could be identified in normal thyroid control tissues (**Figure 6B**).

To test the role of SLC7A11 in PTC, SLC7A11-knockdown TPC-1 and SLC7A11’s over-expressed KTC-1 cell lines were first formulated and tested by western blot (**Figure 6C**). The CCK-8 assay demonstrated that the silence of SLC7A11 would inhibit cell growth and viability (**Figure 6D**). In addition, the loss of SLC7A11 resulted in the suppression of the migration and invasion abilities of PTC cells. Through cell scratch experiments, it was found out that the proliferation rate of SLC7A11 over-expression cells increased (**Figure 6E**). Transwell migration and invasion experiments also proved the ability of SLC7A11 overexpression to promote the migration and invasion of papillary thyroid cancer cells, while SLC7A11 knockdown cells showed the adverse effect (**Figure 6F**).

### Ferroptosis Plays a Crucial Role in Papillary Thyroid Carcinoma and FTO Regulates PTC Cell Ferroptosis by Mediating the m6A Methylation of SLC7A11

The latest research demonstrates that SLC7A11 can enhance tumor development by inhibiting ferroptosis and other ferroptosis-independent functions [31]. Ferroptosis, as an iron-dependent form of regulated cell death, is the result of the lethal accumulation of lipid-based reactive oxygen species (ROS). Then, given the strict regulation of iron homeostasis for the benefit of health, it is unsurprising that disturbances in iron balance would lead to various diseases. It is under speculation that the regulation of SLC7A11 gene expression may be a crucial target of FTO in modulating PTC cell ferroptosis and tumor progression. In our next set of experiments, the role of ferroptosis in PTC is to be investigated.

**Figure 7:**
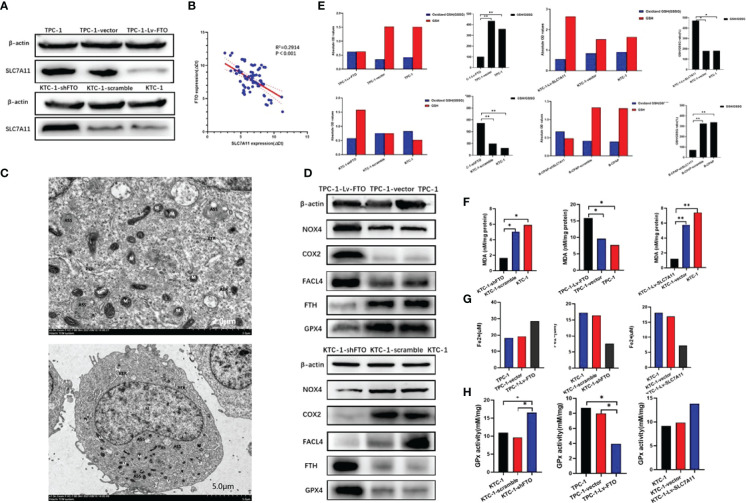
Ferroptosis plays an important role in papillary thyroid carcinoma. Fat mass and obesity-associated protein (FTO) regulates papillary thyroid carcinoma (PTC) cell ferroptosis by mediating the m6A methylation of SLC7A11. **(A)** SLC7A11 protein expression was examined by western blot in TPC-1-Lv-FTO, TPC-1-vector, TPC-1, KTC-1-shFTO, KTC-1-scramble, and KTC-1 cell. **(B)** The mRNA expression of FTO and SLC7A11 in PTC tissue specimens was analyzed by Spearman rank correlation coefficient. **(C)** Ultrastructure of the mitochondria in TPC-1-Lv-FTO cells as imaged by TEM. Red arrowheads, shrunken mitochondria. Scale bars, 2.0 and 5.0 μm. The cells were ovoid, the cell membrane was intact and continuous, a few pseudopods were visible around, the cytoplasm was not obviously edematous, the nucleus was regular, roughly ovoid, and the chromatin was evenly distributed. The mitochondria were fixed, the matrix density was increased, and the cristae were obviously dilated, showing signs of ferroptosis. The rough endoplasmic reticulum was mildly dilated and locally degranulated. A few autophagic lysosomes were visible intracellularly. **(D)** NOX4, COX2, ACSL4, GPX4, and FTH as ferroptosis biomarkers were measured in control and TPC-1-Lv-FTO/KTC-1-shFTO cells by western blot. **(E)** The glutathione (GSH)/oxidized GSH ratio was measured in the indicated PTC cells; *P < 0.05, **P < 0.001. **(F)** Malondialdehyde (MDA) analysis for the evaluation of levels of oxidative stress. MDA was measured in the indicated PTC cells; *P < 0.05, **P < 0.001. **(G)** Fe^2+^ was measured in the indicated PTC cells; *P < 0.05, **P < 0.001. **(H)** GPx was measured in the indicated PTC cells; *P < 0.05, **P < 0.001.

Firstly, SLC7A11 expression in PTC cells was estimated. It is to be noted that SLC7A11 expression was noticeably downregulated in TPC-1-Lv-FTO cells. However, SLC7A11 expression was significantly elevated in KTC-1-shFTO cells (**Figure 7A**). These results demonstrated that FTO might be involved in the regulation of PTC cell ferroptosis. Furthermore, with regard to the mRNA level of FTO and SLC7A11 in 86 pairs of PTC tissues assessed by Spearman rank correlation coefficient analysis, a negative correlation between FTO and SLC7A11 was found (*p* < 0.001; **Figure 7B**).

To clarify the mechanisms in which FTO regulates PTC cell death and tumor progression, the type of cell death upon FTO overexpression was first identified. Furthermore, after the preparation of TPC-1-Lv-FTO cells, TEM was completed at ×2,000 magnification. The cell morphology was found to change significantly as the cell mitochondria became smaller, the membrane density increased, and the cristae decreased sharply or even vanished (**Figure 7C**).

Then, the protein levels of ferroptosis biomarkers—NOX4, COX2, FACL4, and FTH—were set by western blot to detect whether ferroptosis was related to PTC. The results demonstrated that the protein expression of acyl-CoA synthetase long-chain family member 4 (ACSL4) NOX4 and COX2 got markedly elevated, while the GPX4 and FTH levels were overtly reduced in TPC-1-Lv-FTO compared with that of the vector group. The results have also illustrated that the protein expression of ACSL4 NOX4 and COX2 was markedly reduced, while the level of GPX4 and FTH was notably elevated in KTC-1-shFTO in comparison with that of the vector group (**Figure 7D**).

Through monitoring of the levels of MDA (**Figure 7F**), the GSH/GSSG ratio (**Figure 7E**), GPx (**Figure 7H**), and iron ions in various cell lines, as for Fe^2+^ level (**Figure 7G**), MDA in TPC-1-Lv-FTO was found to be significantly higher than TPC-1/vector, and MDA in KTC-1-shFTO proved to be significantly lower than in KTC-1/scramble. In terms of the GSH/GSSG ratio, the GPx level in TPC-1-Lv-FTO was considerably lower than that of the TPC-1/vector, while for GSH/GSSG ratio, the GPx level in KTC-1-shFTO was much higher than that of the KTC-1/scramble. However, the Fe^2+^ level held the opposite trend as MDA in TPC-1-shSLC7A11 was significantly higher than TPC-1/scramble, and MDA in KTC-1-Lv-SLC7A11 was substantially lower than in the KTC-1/vector. When it comes to the GSH/GSSG ratio, the GPx level in TPC-1-shSLC7A11 was remarkably lower than in the TPC-1/scramble, while at the GSH/GSSG ratio, the GPx level in KTC-1-Lv-SLC7A11 was dramatically higher than in the KTC-1/vector.

### The Effects of FTO Inhibition Are Reversed by the Loss of SLC7A11

**Figure 8:**
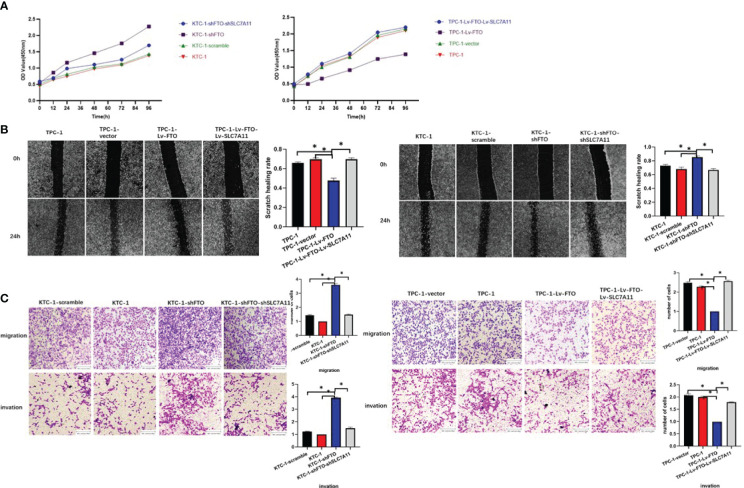
The effects of fat mass and obesity-associated protein inhibition are reversed by the loss of SLC7A11. **(A)** CCK-8 assay. **(B)** Scratch test. **(C)** Transwell migration and invasion assay. *, P<0.05.

Finally, whether FTO could regulate PTC cell ferroptosis by mediating the m6A demethylation of SLC7A11 was reversely verified. To validate that the observed phenotype was mediated by the dysregulation of the FTO–SLC7A11 axis, a few functional rescue assays were performed. Then, as demonstrated by the CCK-8 assay, the knockdown effect of FTO resulted in the enhanced proliferation capacity in KTC-1 cells, which could be reverted by SLC7A11-knockdown. The over-expression of SLC7A11 also substantially increased the abolished mobility induced by the over-expression of FTO (**Figure 8A**). Besides this, wound healing assays (**Figure 8B**) and transwell migration and invasion assays (**Figure 8C**) also confirmed the initial speculation. To sum up, SLC7A11 dysfunction may lead to FTO-mediated PTC cell proliferation or mobility signatures.

## Discussion

Mounting evidence has shown that m6A modification is critical to mitigating the progression of various cancers, including endometrial cancer (23), gastric cancer (21), hepatocellular carcinoma (27), bladder cancer (28), lung cancer (20), and acute myeloid leukemia (29).

In the human mRNA level, m6A is considered to be the most enriched internal chemical modification. Thanks to the availability of m6A-specific antibodies and the state-of-the-art high-throughput sequencing technology, researchers are now able to precisely determine the exact m6A sites and further study on its functions in both pathological and biological processes (30). Increasing evidence has shown the correlation between m6A modification and a variety of solid tumors (31, 32); however, the lack of research on the distribution of m6A and its potential functions and mechanisms in PTC remains a challenge. In this study, FTO mRNA levels were found to be substantially downregulated in PTC tissues compared with matched adjacent normal tissues. Overall, our findings are consistent with a prior cytological study in PTC cells (32), even though they are contrary to the increased FTO expression in some other types of cancer as extrapolated from other investigations (33, 34).

Demethylases are required for the removal of reversible m6A modifications, and FTO is the first protein confirmed to catalyze m6A demethylation (5). FTO ensures the balance of m6A modification in the transcriptome and is widely recognized for its role in human obesity and adipogenesis. Meanwhile, it is also correlated with mitochondrial biogenesis and oxidative stress due to its capacity to modify associated genes post-transcriptionally (35). On account of its role in cellular metabolism, FTO is regarded as a potential factor in the pathogenesis of a variety of cancers by inducing chemoresistance and tumorigenesis (36, 37). Chen and He’s team displayed the carcinogenic effect of FTO in acute myeloid leukemia (38). Elevated FTO levels are also identified in glioblastoma and cervical squamous cell carcinoma (39). The inactivation of FTO in leukemia causes the drug-resistant cells to become sensitive to tyrosine kinase inhibitors (40), suggesting that the FTO–m6A axis may serve as a new therapeutic target for human cancers. The potential epigenetic regulation of FTO in the incidence and progression of PTC is still being investigated. In this study, we aim to clarify the role of m6A modification in PTC as well as to study the underlying molecular mechanism by which m6A modification affects the incidence and progression of PTC.

In order to further clarify the effects of FTO on the proliferative activity and invasive responses of PTC cells, FTO overexpression and knockdown cell lines were generated. Through CCK-8, scratch test, transwell migration, invasion experiments, apoptosis tests, and tumor formation tests in nude mice model, over-expression of FTO was reported to reduce the tumor growth of PTC, cell proliferation, migration, and invasion. Conversely, FTO knockdown facilitates PTC progression. As a result, these findings suggest that FTO may function as a PTC tumor suppressor.

Despite the fact that understanding regarding the specific mechanisms of FTO in PTC remains limited, the expression of FTO in PTC has been detected as being downregulated. The hypothesis was that FTO may play distinct functions in various tumor types as part of a complex tumorigenesis mechanism. Referring to the collected information on the clinicopathological features of patients suffering from papillary thyroid carcinoma, a qRT-PCR analysis was conducted on clinical samples to evaluate whether differences existed in the expression of FTO and SLC7A11 in tumor tissues and corresponding non-tumor normal tissues. Furthermore, our immunohistochemical staining results verified the low expression of FTO in tumor tissues.

Next, through RNA-seq and MeRIP-seq analyses, SLC7A11 was identified as a downstream target of FTO-mediated m6A modification, which was further confirmed by luciferase and western blot analysis in control and FTO over-expression/knockout PTC cells. Our results show that FTO inhabitation can cause PTC malignancies by weakening the expression of SLC7A11, which implies that the impact of FTO on carcinogenesis is contingent on different downstream molecules and the specific tissue environment. The findings in this paper emphasize that m6A demethylase FTO plays an inhibitory role in the progression of PTC. The over-expression of FTO-mediated m6A demethylation inhibits the expression of SLC7A11, thus facilitating ferroptosis in PTC and suppressing the growth of thyroid cancer. FTO knockdown significantly curbs the expression of ferroptosis in PTC cells while also promoting tumor progression. These findings clarify the inhabitation effect of FTO on the malignancy of PTC through lowering the SLC7A11 expression. This indicates that the effects of FTO on carcinogenesis depend on the specific tissue environment and various downstream molecules as well.

As an amino acid antiporter, SLC7A11 is in charge of extracellular cysteine uptake in exchange for glutamate. The reduction of SLC7A11 leads to the imbalance of glutathione biosynthesis and cysteine uptake, resulting in glutathione peroxidase 4 inhibition and iron ptosis activation (41). The SLC7A11 subunit in the cystine/glutamate antitransport system takes charge of the internalization of intracellular glutamate and extracellular cystine to produce glutathione for clearance lipid ROS (42–44). The correlation between SLC7a11 and FTO was examined in regard to the crucial involvement of SLC7A11 in system Xc- and ferroptosis. It was speculated that FTO may regulate PTC ferroptosis death through direct mediation of m6A demethylation in the ferroptosis suppressor gene SLC7A11.

Recent studies have clearly testified the potential mechanism and application value of ferroptosis in tumor treatment. Ferroptosis, proposed in 2012 by Stockwell BR lab, has been conceived as a necrotic cell death modality different from necrosis, apoptosis, biochemistry, and genetics and autophagy in morphology (45). The buildup of reactive oxygen species (45) defines the hallmark of such process *via* an iron-dependent mechanism. Cysteine depletion, which results in the intracellular exhaustion of glutathione, serves as a preliminary feature of the mechanism and triggers ferroptosis (45). Hence, it is conceivable that the complex interaction regulating the sensitivity of various cancer cells to ferroptosis should be a luxuriant field of cancer research. A great number of studies have demonstrated that many genes are involved in the occurrence and treatment of ferroptosis in cancers (46). These findings shed light on tumor ferroptotic plasticity and propose insights into the correlation between ferroptosis and the de-differentiation, persistence, and expansion of cancer cells, respectively.

The biological features of ferroptosis are distinguished by the aggregation of iron and ROS, which reduces the level of cystine uptake and consumes GSH to inhibit the activities of system xc- and GPX4. Moreover, maintaining a stable intracellular GSH concentration can protect the cells from ferroptosis and oxidative stress responses. On top of that, research on system xc-mediated cystine uptake has demonstrated its significance for glutathione synthesis, which appears to be a key mechanism for preventing lipid peroxidation and suppressing ferroptosis (47). Ferroptosis, as a new form of programmed and no-apoptosis cell death, would be triggered by iron-dependent lipid peroxidation (48). Lipid peroxidation has been analyzed by the quantification of MDA levels. The data in this paper demonstrates that TPC-1-Lv-FTO cells reduce the GSH levels, thus triggering ferroptosis.

Western blot was used to detect the molecular markers of ferroptosis, such as NOX4, COX2, FACL4, and FTH. Lipid peroxidation and iron metabolism signals were considered as the key mediators of ferroptosis. The iron oxidase activity of FTH catalyzes the conversion of the ferrous form (Fe^2+^) to the ferric form (Fe^3+^) and facilitates the safe integration of iron into the ferritin shell, thus cutting down the level of free iron (49). Furthermore, lipid peroxidation is affected by several lipids and enzymes, a genome-wide CRISPR-based gene. Screening and microarray analysis of ferroptosis-resistant cell lines reveal that ACSL4 serves as the fundamental component for executing ferroptosis (50). Many studies have shown that GPX4, as the main target of ferroptosis, promotes the reduction of intracellular lipid peroxide under the ferroptosis condition (44, 51). A recent study has demonstrated that the suppression of FTH1 would increase the incidence of ferroptosis, indicating the likelihood of FTH1’s presence in ferroptosis and suggesting that interruption of iron storage may result in iron overload during ferroptosis (52). Evidence shows the correlation between NOX4 and hydrogen peroxide in promoting cell death, and the inhibition of NOX4 can obstruct ferroptosis of the renal tubular epithelium (53). NOX4-induced iron ptosis-dependent cytotoxicity is enhanced by activating lipid peroxidation in human astrocytes (48).

It is worth noting that, although the downstream targets of FTO have been researched as mentioned above, studies on the upstream subject of FTO remain deficient. Therefore, possible inducive factors of FTO deficiency in PTC have been explored. Regulating the expression of SLC7A11 gene is believed to be a critical, but not the sole, target for FTO to regulate tumor progression and ferroptosis in PTC cells. Whether FTO also regulates other target genes associated with PTC malignancies requires experiments for verification.

In this study, FTO has been found to be downregulated in PTC and functionally inhibits the proliferation and invasiveness of PTC cells *in vivo* and *in vitro*. By inhibiting the m6A modification of SLC7A11, FTO downregulated the SLC7A11 expression. In conclusion, this work demonstrates the critical role of FTO overexpression in PTC progression and the validity of a novel epigenetic regulatory mechanism through which FTO regulates PTC ferroptosis by downregulating the ferroptosis suppressor gene SLC7A11 through m6A demethylation, thereby inhibiting the development of PTC. In short, targeting FTO may be a new approach to achieve therapeutic PTC treatment.

## Conclusions

Overall, convincing evidence has been provided to confirm that FTO suppresses PTC metastasis by downregulating the expression of SLC7A11, which indicates that FTO might work as a favorable predictor for PTC.

In conclusion, this paper reveals the tumor-suppressive properties of FTO in the development of PTC. The downregulation of FTO activates the m6A mechanism and contributes to the epigenetic activation of SLC7A11. These results suggest that FTO may be an ideal predictor of PTC, highlight the attractive value of m6A demethylase in enhancing the understanding of m6A epitranscriptomic modification in papillary thyroid carcinoma research, and, more importantly, suggest potentials of developing effective predictors and treatment strategies of PTC. Future studies are expected to explore deeply into the prospects of m6A-regulated tumor malignancies and acquire new insights of potential biomarkers and even therapeutic targets for PTC therapy.

## Data Availability Statement

The data presented in the study are deposited in the GEO database repository, accession number GSE199207.

## Ethics Statement

The studies involving human participants were reviewed and approved by 2021-KY-0202-002, Ethics Committee of the First Affiliated Hospital of Zhengzhou University. The patients/participants provided their written informed consent to participate in this study.

## Author Contributions

F-HJ contributed to the conception and study design. F-HJ and X-HF conducted the experiment and collected data. F-HJ, X-HF, and G-QL analyzed the data. F-HJ, G-QL, and QH drafted the manuscript. X-GQ was responsible for revising and correcting the manuscript. All authors contributed to the article and approved the submitted version.

## Funding

This work was supported by a grant from Thermal Ablation of Thyroid Nodules International Joint Laboratory [Henan Province; YUKEWAI (2016), number 11]. We acknowledge assistance with the access of analytic instruments from Translational Medical Center at The First Affiliated Hospital of Zhengzhou University.

## Conflict of Interest

The authors declare that the research was conducted in the absence of any commercial or financial relationships that could be construed as a potential conflict of interest.

## Publisher’s Note

All claims expressed in this article are solely those of the authors and do not necessarily represent those of their affiliated organizations, or those of the publisher, the editors and the reviewers. Any product that may be evaluated in this article, or claim that may be made by its manufacturer, is not guaranteed or endorsed by the publisher.
